# Factors associated with stigma attitude towards people living with HIV among general individuals in Heilongjiang, Northeast China

**DOI:** 10.1186/s12879-017-2216-0

**Published:** 2017-02-17

**Authors:** Xin Li, Lili Yuan, Xiaoxia Li, Jingli Shi, Liying Jiang, Chundi Zhang, Xiujing Yang, Yeli Zhang, Donghui Zhao, Yashuang Zhao

**Affiliations:** 1 Department of Epidemiology, Public Health College, Harbin Medical University, Harbin, Heilongjiang Province China; 2 Harbin Medical University, Harbin, Heilongjiang Province China; 3 Heilongjiang Medical Science Institute, Harbin, Heilongjiang Province China; 4 Department of Public Health, Mudanjiang Medical College, Mudanjiang, Heilongjiang Province China; 5 Qiqihaer Center for Disease Control and Prevention, Qiqihaer, Heilongjiang Province China; 6 Department of Epidemiology, Public Health College, Nantong University, Nantong, Jiangsu Province China; 7 Qiqihaer Medical University, Qiqihaer, Heilongjiang Province China; 8 Clinical Laboratory, Third Affiliated Clinical Hospital of Qiqihaer Medical University, Qiqihaer, Heilongjiang Province China; 9 Disease Control Office, Health Department of Heilongjiang Province, Harbin, Heilongjiang Province China; 10 Heilongjiang Center for Disease Control and Prevention, Harbin, Heilongjiang Province China

**Keywords:** HIV/AIDS knowledge, General individuals, Public stigma attitude

## Abstract

**Background:**

HIV-related stigma always is major obstacles to an effective HIV response worldwide. The effect of HIV-related stigma on HIV prevention and treatment is particularly serious in China. This study was to examine stigma attitude towards people living with HIV/AIDS (PLWHA) among general individuals in Heilongjiang Province, Northeast China and the factors associated with stigma attitude, including socio-demographic factors and HIV/AIDS Knowledge.

**Methods:**

A cross-sectional survey was carried out in Heilongjiang Province, China. A total of 4050 general individuals with age 15–69 years in four villages in rural areas and two communities in urban areas were drawn using stratified cluster sampling. Standardized questionnaire interviews were administered. Univariate and multivariate log-binomial regression were performed to assess factors affecting stigma attitude towards PLWHA.

**Results:**

The proportions of participants holding stigma attitude towards PLWHA were 49.6% among rural respondents and 37.0% among urban respondents (*P* < 0.001). Multivariate log binomial regression analysis among both rural participants (RR = 0.89, 95% CI: 0.87–0.91, *P* < 0.001) and urban participants (RR = 0.89, 95% CI: 0.87–0.91, *P* < 0.001) showed that greater knowledge of HIV transmission misconceptions was significantly associated with lower stigma attitude towards people living with HIV. And among urban participants, higher education level (high school vs. primary school or less: RR = 0.73, 95%CI: 0.62–0.87, *P* < 0.001; middle school vs. primary school or less: RR = 0.83, 95%CI: 0.71–0.97, *P* = 0.018) were also significantly associated with lower stigma attitude towards PLWHA.

**Conclusions:**

The level of stigma attitude towards PLWHA is higher in rural areas than in urban areas in Heilongjiang. Meanwhile, individuals who better were aware of HIV/AIDS transmission misconceptions may hold lower stigma attitude toward PLWHA whether among rural or urban residents.

## Background

China is experiencing a rapid growth in the number of people living with HIV, and the epidemic of HIV/AIDS is spreading from high risk population to the general individuals. It was estimated that approximately 840,000 Chinese were HIV infected by the end of 2013, whereas the cumulative number of HIV infections actually diagnosed was 434,000 in China, and 48.3% of HIV carriers were not aware of their infection [1]. Heilongjiang Province Centers for Disease Control and Prevention reported the incidence has increased 14.69 times from 2004 (0.16 per 100,000) to 2013 (2.35 per 100,000) even in Heilongjiang Province, an area with low HIV prevalence, northeast China.

HIV stigma is one of the key social factors expanding HIV prevalence and hindering HIV preventive and treatment [2]. Numerous studies have found HIV-related stigma was related with delayed HIV testing, and poor engagement with HIV services and nondisclosure to sexual partners [3–6]. Consistent with other countries, currently HIV infection is a highly stigmatised disease in China [7, 8]. The effect of HIV-related stigma on HIV preventive and cure is especially prominent in China. PLWHA who know their status avoid treatment services [9] and hide their sera-status status from partners [10–12].

In China, studies about stigma attitude towards PLWHA primarily focused on high-risk groups such as rural-to-urban migrants [13], female migrants [14], commercial blood donors [10, 15, 16], and university students [17]. There have been few studies among the general individuals about factors associated with stigma attitude towards PLWHA in China [18, 19]. Although studies have shown that HIV related stigma attitudes are largely fuelled by ignorance about HIV transmission [20, 21], findings from these foreign studies may not be applicable in general population in China because of the multifaceted and complexity nature of stigma attitude.

HIV-related stigma attitude is particularly obvious in both self-stigma and public stigma. Self-stigma is the individual’s perception that he or she is socially unacceptable, while public stigma is society’s perception that an individual is socially unacceptable [22–24]. Public stigma is the focus of this study, because it is closely related with HIV prevention and treatment [25]. The purpose of this paper is to evaluate the level of stigma attitude towards PLWHA and factors associated with the stigma attitude towards PLWHA among general individuals in Heilongjiang, Northeast China. The findings of the study point out the ways of decreasing stigma attitude towards PLWHA in both urban and rural areas of Heilongjiang Province.

## Methods

### Participants and procedures

The study design was described as previously [26]. In brief, a total of 4050 general individuals with age 15–69 years were drawn from rural (September 2007) and urban areas (April 2008) using stratified sampling methods. First, two cities, Qiqihaer and Mudanjiang representing a middle socioeconomic level in urban areas (The population density is relatively high, usually more than 100,000 people; people mainly engage in non-agricultural industries), were selected from a total of seven cities. Then two communities of a middle socioeconomic level were selected respectively in the two cities, and all residents of selected communities were invited to participate. Fuyu and Dongning counties with a middle socioeconomic level in rural areas (The population density is relatively scarce; people mainly engage in agricultural production) was selected. Then four villages with a middle socioeconomic level were selected respectively in Fuyu and Dongning counties, and all residents of selected four villages were invited to participate. After providing informed consent, trained interviewers went door-to-door to invite people and participants complete an anonymous questionnaire in a separate room at home. The interviewers provided assistance to some individuals with limited literacy by reading the questionnaire. It took about 15 min for participants to complete the questionnaire. Finally, 4002 were recruited and complete the survey, and the response rate was 98.8%. The study protocol was approved by the Committee on Human Research of Harbin Medical University.

### Measures

The questionnaire consisted of demographic information, the 15 HIV -related knowledge questions [27, 28], three questions about stigma attitude to reflect public stigma [28] and one question about willingness to participate in a free HIV test. Factors associated with willingness to participate in free HIV test were analyzed in the previous paper [26], meanwhile the HIV-related knowledge questions were described in detail. The three questions about stigma attitude included whether participants were willing to work with people with HIV/AIDS, to accept family members with HIV/AIDS, or have their children to study with people with HIV/AIDS. We defined “having stigma attitude towards PLWHA” if participants gave a “stigma response” to any of those three questions.

### Statistics analysis

The Student t-test or Chi-square test was applied to evaluate the differences of knowledge and stigma attitude towards PLWHA between urban participants and rural participants. Univariate log binomial regression was applied to examine the associations between each variable and stigma attitude towards PLWHA. Variables in the univariate analysis with a significance level (*P* < 0.10) were entered into the multivariate analysis. Multivariate log binomial regression was used to identify factors influencing stigma attitude, adjusted for demographic characteristics (i.e. gender, age,). These data of urban participants and rural participants were analyzed separately owing to great economic and cultural differences between rural areas and urban areas in China. Data were entered with Epidata 3.02 and analysed using SAS Software 9.1.

## Results

The proportion of participants holding stigma attitude towards PLWHA among rural respondents (49.6%) was significantly higher than that among urban respondents (37.0%; *P* < 0.001). And 33.8% of rural respondents and 26.3% of urban respondents thought that people with HIV should be kept away from their colleagues (*P* < 0.001); 10% of rural and 9.6% of urban respondents were not willing to accept family members with HIV/AIDS (*P* = 0.706); and 33.8 and 23.0%, respectively, would not agree their children to study with PLWHA (*P* < 0.001).

Factors associated with stigma attitude towards PLWHA identified with univariate and multivariate log binomial regression analyses in urban participants and rural participants were shown in Table 1. Results of multivariate log-binomial regression analyses in urban respondents adjusted for gender, age, marital status, education, income and employment showed a negative relationship between education levels and stigma attitude towards PLWHA. Specifically, compared with primary school or less, middle school education was associated with a 17% reduction in stigma attitude toward PLWHA (*P* =0.018), while high school education was associated with a 27% reduction (*P* < 0.001). Stigma attitude towards people with HIV was 30% lower in divorced or widowed persons than in single persons. However for rural respondents, age was the only significant socio-demographic predictor. Being 21–50 years of age was associated with a 26% decrease in stigma attitude toward people with HIV (*P* = 0.038). The results also revealed significant relation between HIV related knowledge and stigma attitude towards PLWHA. Specifically, having greater knowledge of HIV transmission misconceptions was significantly associated with a 12% decrease in stigma attitude towards PLWHA in urban respondents (*P* < 0.001). In contrast, being aware that AIDS as a contagious disease was associated with a significant 45% increase in the odds of stigma attitude towards people with HIV in urban respondents (*P* < 0.001). These findings were consistent with what the data showed for rural respondents. Having better knowledge of HIV transmission misconceptions was significantly associated with an 11% decrease in stigma attitude towards people with HIV among rural respondents (*P* < 0.001). And being aware that AIDS was a contagious disease was significantly associated with a 58% increase in stigma attitude toward people with HIV (*P* < 0.001). Whereas having better knowledge of HIV transmission modes made no difference for urban participants, it was significantly associated with a 6% increase in stigma attitude toward people with HIV for rural participants (*P* = 0.003).

**Table 1:** Univariate and multivariate log binomial analysis of factors associated with stigma attitude toward people with HIV

		Urban residents		Rural residents	
Variable		Crude RR	Adjusted RR	Crude RR	Adjusted RR
Gender	Male	1.00	1.00	1.00	1.00
	Female	0.95 (0.85–1.07)	0.98 (0.88–1.09)	1.01 (0.92–1.11)	0.97 (0.88–1.06)
Ethnicity	Han	1.00	1.00	1.00	1.00
	Minorities	1.15 (1.00–1.33)^*^	1.09 (0.94–1.26)	0.940.84–1.06)	0.95 (0.85–1.06)
Age (Years)	15–20	1.00	1.00	1.00	1.00
	21–50	0.88 (0.72–1.09)	1.02 (0.81–1.3)	1.09 (0.90–1.31)^**^	1.26 (1.01–1.57)^*^
	51–69	0.87 (0.69–1.10)	0.95 (0.73–1.25)	1.01 (0.82–1.23)^**^	1.12 (0.88–1.42)
Marital status	Single	1.00	1.00	1.00	1.00
	Married/cohabitating	0.91 (0.80–1.05)	0.96 (0.82–1.12)	0.97 (0.85–1.10)	0.92 (0.80–1.06)
	Divorced/widowed	0.80 (0.57–1.10)	0.70 (0.50–0.99)^*^	0.75 (0.48–1.16)	0.75 (0.49–1.13)
Education	Primary school or less	1.00	1.00	1.00	1.00
	Middle school	0.77 (0.65–0.91)^**^	0.83 (0.71–0.97)^*^	0.95 (0.87–1.05)	0.96 (0.87–1.06)
	Over high school	0.62 (0.53–0.73)^**^	0.73 (0.62–0.87)^**^	0.93 (0.78–1.11)	1.02 (0.84–1.23)
Employment	Unemployed	1.00	1.00	1.00	1.00
	Employed	1.25 (1.00–1.57)	1.28 (1.02–1.60)*	0.96 (0.84–1.11)	0.96 (0.82–1.12)
Household per-capita income (CNY/month)	<1000	1.00	1.00	1.00	1.00
	1000–2000	0.94 (0.81–1.09)	1.04 (0.89–1.20)	0.97 (0.86–1.08)	0.95 (0.85–1.06)
	≥2000	1.08 (0.91–1.29)	1.16 (0.98–1.38)	0.97 (0.83–1.13)	0.92 (0.79–1.06)
HIV/AIDS Knowledge					
Is AIDS a contagious disease?		1.12 (0.95–1.31)	1.45 (1.23–1.71)^**^	1.47 (1.27–1.71)^**^	1.58 (1.34–1.86)^**^
Can an apparently healthy person be a carrier for HIV?		0.78 (0.69–0.87)^**^	0.94 (0.81–1.09)	1.01 (0.92–1.11)	0.99 (0.89–1.10)
At present, is there a vaccine to protect against HIV?		0.71 (0.63–0.79)^**^	0.88 (0.77–1.00)	0.92 (0.83–1.01)	0.94 (0.84–1.04)
At present, is AIDS curable?		0.70 (0.63–0.79)	0.89 (0.79–1.02)	0.94 (0.86–1.03)	1.01 (0.91–1.11)
HIV/AIDS transmission modes score		0.91 (0.88–0.94)^**^	1.01 (0.96–1.05)	1.03 (1.00–1.06)	1.06 (1.02–1.09)^**^
HIV/AIDS transmission misconceptions score		0.86 (0.84–0.89)^**^	0.88 (0.85–0.91)^**^	0.94 (0.92–0.96)^**^	0.89 (0.87–0.91)^**^
Total knowledge score		0.94 (0.93–0.95)^**^		0.99 (0.98–1.00)^*^	

Crude RR: Relative risk of univariate analysis

Adjusted RR: Basic demographic characteristics (i.e., age, gender) and variables that were associated with stigma attitude toward people with HIV in univariate analysis among general residents at the 0.10 significance level were put stepwise in a multivariate logistic regression model

*RR* Relative risk (95% CI)

^*^ *P* <0.05; ^**^ *P* <0.01

## Discussion

Although antiretroviral therapy (ART) scale-up may reduce HIV-related stigma, declines in social distancing seemed to be more pronounced in countries with high HIV prevalence [29]. It suggest that ART scale-up may be beneficial for stigma reduction but is unlikely to be a panacea, especially in countries with relatively low HIV prevalence. The purpose of this paper is to evaluate the level of stigma attitude towards PLWHA and factors associated with stigma attitude towards PLWHA among general individuals in Heilongjiang, an area with low HIV prevalence, northeast China. In our study, the proportions of participants holding stigma attitude towards people living with HIV were 37.0% among urban residents and 49.6% among rural residents (*P* < 0.001). The levels of stigma attitude towards PLWHA were similar with Laurie Able’s study in Guangxi [19], Derlega’s study in a province in Southwestern China [30] and Chen Jiajian’s study in 7 provinces in China [18], in which the levels of stigma were 40, 52.9 and 45%, respectively. These findings from different areas of China suggested that stigma attitude towards PLWHA is prevalent in both high-risk areas and low-prevalence areas in China. Furthermore, we found rural participants are more likely to hold stigma attitude towards PLWHA compared to urban participants. It may be due to the lack of effective publicity and education on HIV/AIDS in rural areas in Heilongjiang Province.

The multivariate log-binomial regression analysis showed that the older participants in rural areas are more likely to hold stigma attitude towards PLWHA, and not in urban areas. This finding is consistent with J T F Lau’s study in Hong Kong [8, 30]. However it is different with Letamo G’s study in Botswana [21] with the high prevalence of HIV, which young people more likely had discriminatory attitude towards people with HIV/AIDS. The multivariate log-binomial regression analysis also showed that the urban participants with lower level of education more likely hold stigma attitude towards PLWHA. Other studies found similar findings that stigma was more common among those with lower level of education [8, 18]. The multivariate log-binomial regression analysis among both urban participants and rural participants showed that the individuals who were aware of AIDS being a contagious disease were more likely to hold stigma attitude towards PLWHA. The evidence suggests stigma attitude towards PLWHA may be driven by the fear of infection. Caroline Kingori’s study in rural Central Kenya [31] and J T F Lau’s study in Hong Kong [30] showed that increased stigma and discrimination are associated with low levels of HIV transmission knowledge. In our study, increased stigma attitude are associated with better knowledge of HIV transmission modes for rural participants, not significantly associated for urban participants. However, the individuals who better were aware of HIV/AIDS transmission misconceptions may hold lower stigma attitude towards PLWHA whether among rural or urban participants. The evidence suggests stigma attitude toward PLWHA may be due to overestimate the risk of HIV contagion, and avoid contact with PLHIV. This result is similar to Chen Jiajian’s study among men and women who were aged 15-49 [18], Laurie Abler’s study in a city in southwest China with high HIV prevalence [19], J T F Lau’s study in Hong Kong [8] and Letamo G’s study in Botswana [22]. Moreover, our study findings also revealed that there was a high level of misunderstanding about the six common HIV transmission among the population, only less than one fifth of the urban participants and one tenth of the rural participants correctly answered all six questions about HIV transmission misconceptions, while nearly half of the urban participants and two fifths of the rural participants correctly answered all five questions about HIV transmission modes. This finding is consistent with the study in southwest China in 2003 [32] and the study in Xinjiang in 2006 [33]. It indicates that the effectiveness and feasibility of the current education programs are still limited, or the education about how HIV is not transmitted is urgent needed.

Overall, these findings provide valuable information for promoting HIV stigma prevention strategies in Heilongjiang Province. This study examined the factors associated with stigma attitude among general individuals in Heilongjiang, including demographic characteristics and HIV/AIDS Knowledge. The findings highlight that misconceptions about HIV transmission modes should be addressed in HIV prevention and education programs, and effective education programs should be advanced, for example, professional explanation about why AIDS is not transmitted through casual contacts by scientists and authoritative medical experts. In addition, the HIV stigma prevention programs should be carried out preferentially, which target populations are the older residents in rural areas and the urban residents with lower education level in Heilongjiang.

However, our study has some limitations that should be considered. First, this was a cross sectional study. Second, other parts of the stigma attitudes was not measured, such as self-stigma attitude. Third, our questionnaire lacked information on tuberculosis and high risk behaviors. Finally, as the samples were not perfect random samples, some selection bias may exist. Despite these limitations, the study results provide evidences for a follow-up long-term study and intervention.

## Conclusions

Our results indicate that the level of stigma attitude towards PLWHA is higher in rural areas than in urban areas in Heilongjiang. Therefore, further studies are needed to find effective ways to decrease stigma attitude towards PLWHA among rural individuals. Our results indicate that the urban individuals with lower levels of education and the older rural individuals may hold higher stigma attitude toward PLWHA. Meanwhile, the individuals who better were aware of HIV/AIDS transmission misconceptions may hold lower stigma attitude toward PLWHA whether in urban or rural areas of Heilongjiang Province. Therefore, there is a need to effective intervention programs in Heilongjiang that focus on raising aware of HIV/AIDS transmission misconceptions among the general individuals, especially, the urban individuals with lower levels of education and the older rural individuals, and measure changes in the stigma attitude toward PLWHA.

## Abbreviations

AIDS: Acquired immunodeficiency syndrome
ART: Antiretroviral therapy
HIV: Human Immunodeficiency Virus
PLWHA: People living with HIV/AIDS
